# Frontier battery development for hybrid vehicles

**DOI:** 10.1186/1752-153X-6-S1-S2

**Published:** 2012-04-23

**Authors:** Heather Lewis, Haram Park, Maion Paolini

**Affiliations:** 1 New York University School of Law, J.D., expected 2013; USA; 2 Ecole Polytechnique, Palaiseau, France

## Abstract

**Background:**

Interest in hybrid-electric vehicles (HEVs) has recently spiked, partly due to an increasingly negative view toward the U.S. foreign oil dependency and environmental concerns. Though HEVs are becoming more common, they have a significant price premium over gasoline-powered vehicles. One of the primary drivers of this “hybrid premium” is the cost of the vehicles’ batteries. This paper focuses on these batteries used in hybrid vehicles, examines the types of batteries used for transportation applications and addresses some of the technological, environmental and political drivers in battery development and the deployment of HEVs.

**Methods:**

This paper examines the claim, often voiced by HEV proponents, that by taking into account savings on gasoline and vehicle maintenance, hybrid cars are cheaper than traditional gasoline cars. This is done by a quantitative benefit-cost analysis, in addition to qualitative benefit-cost analysis from political, technological and environmental perspectives.

**Results:**

The quantitative benefit-cost analysis shows that, taking account of all costs for the life of the vehicle, hybrid cars are in fact more expensive than gasoline-powered vehicles; however, after five years, HEVs will break even with gasoline cars.

**Conclusions:**

Our results show that it is likely that after 5 years, using hybrid vehicles should be cheaper in effect and yield a positive net benefit to society. There are a number of externalities that could significantly impact the total social cost of the car. These externalities can be divided into four categories: environmental, industrial, R&D and political. Despite short-term implications and hurdles, increased HEV usage forecasts a generally favorable long-term net benefit to society. Most notably, increasing HEV usage could decrease greenhouse gas emissions, while also decreasing U.S. dependence on foreign oil.

## Background

America is an automobile-oriented nation. In 2007, Americans owned more than 237 million passenger vehicles. Since the advent of the interstate highway system, the number of passenger vehicles in the U.S. has been continuously growing, and the number of vehicles has exceeded the number of registered drivers since 1972. Though fuel economy has improved by almost 40% since 1960, these improvements have not been enough to keep pace with the sheer number of passenger vehicles, which has more than doubled in the last 60 years [1]. An increasingly negative view toward the U.S. foreign oil dependency and environmental concerns has been pushing interest in electric and hybrid-electric vehicles. In 2007, passenger vehicles were responsible for 1,600 MMT of CO_2_ emissions, or more than one-quarter of the total CO_2_ emissions for the US [2].

Consequently, many efforts to reduce U.S. greenhouse gas emissions have focused on reducing emissions from passenger vehicles. HEVs are at the forefront of these reduction efforts. HEVs combine a traditional internal combustion engine with an electric motor and a battery pack, and can reach a fuel economy of 50 miles per gallon (nearly triple the current average miles traveled per gallon for passenger vehicles). There are a few production HEVs that are currently available to consumers, including the Toyota Prius, the Honda Insight and the Honda Civic Hybrid. Plug-in hybrid electric vehicles (PHEVs) have the same components as HEVs, but additionally can be plugged in to an external electric power source to recharge the batteries. PHEVs are far less common on the roads than HEVs, although General Motors, Toyota, Volkswagen, Volvo and Ford all have plans to release PHEVs in the next three years. This report will focus on the more widely-available HEVs, which are currently better situated to become cost competitive with traditional gasoline-powered cars.

Though HEVs are becoming more common in the commercial vehicle fleet, there is a significant price premium on hybrid vehicles over similar gasoline-powered vehicles. One of the primary drivers of this “hybrid premium” is the cost of the vehicles’ batteries. This paper will focus on these batteries used in hybrid vehicles. This paper will examine the types of batteries used for transportation applications and address some of the technological and political barriers to further battery development and deployment in HEVs. Ultimately, this paper will examine the claim, often voiced by HEV proponents, that taking into account savings on gasoline and vehicle maintenance, hybrid cars are cheaper than traditional gasoline cars. A benefit-cost analysis will show that, taking account of all costs for the life of the vehicle, hybrid cars are in fact more expensive than gasoline-powered vehicles; however, after five years HEVs will break even with gasoline cars.

### Battery technologies

The principles upon which batteries operate were established in the nineteenth century, but demand for more efficient and higher capacity batteries has continued to outpace the development of batteries themselves. Batteries have a significant number of limitations that make them difficult to use in cars, and a large part of the potential for battery-powered vehicles is based on the possibility of improvements in battery technology.

In order to understand the limitations of battery development it is important to understand how batteries work. The basic chemical process in batteries has not changed significantly over the past 200 years. A battery’s primary function is to convert chemical energy into a direct current for use in electrical applications. This is accomplished by a reaction between two electrodes connected by an electrolyte in a cell. Through reduction and oxidation, electrons are transferred across the electrolyte from one electrode to another, and a current and voltage is produced. Several of these cells are linked together to form a battery. The materials that make up the electrode and the electrolyte differ depending on the battery, but this chemical reaction is the basis of all currently widely used batteries. The materials used in designing a battery place limits on the maximum energy and power that can be drawn, and additionally affect the charging cycle.

The amount of energy available in a battery is measured in several ways. The charge of a battery is given out in Amphours, or how many amps can flow from a battery per hour. The capacity of a given battery is usually labeled C. (A battery with a 30 Amphour capacity means C=30.) Another measure of a battery’s energy is the specific energy defined as Wh/kg. This is the amount of energy available relative to the weight. Related is the energy density, which is the amount of energy in relation to volume (Wh/L). All of these ratings are important in determining the physical size of a given battery type needed for a given application.

Unfortunately, these parameters can only be defined nominally in most instances. Changes in the temperature and discharge rate greatly affect the energy available to use. This is due to unwanted side reactions that do not transfer the energy in the chemical bonds into useable electricity. When a battery has been discharged significantly, usually below 20%, the efficiency of the reactions sharply decreases as well.

Unwanted reactions also occur spontaneously when the battery is not in use. This leads to what is termed self-discharge. Different batteries have different rates of self-discharge and this has a significant impact on the viability of a battery for use in applications where the battery will go a long time between uses, as is common in private vehicles. This self-discharge also has an impact on recharging efficiency. Though the current across cells while the battery is in use is equal, the self-discharge rate differs because of variance in manufacturing and in temperature across the cells. Some cells discharge at a higher rate than other cells in the battery. Similarly, as the battery is recharged, come cells become “full” before the others. When this happens, these cells must be “over-charged” until charge equalization occurs and all the cells are “full”. This equalization has to occur at a much slower rate than the initial charging in order to avoid damaging the battery and to prevent fire or explosion. The slower rate makes it difficult to quickly recharge batteries to their full capacity.

Currently there are three types of batteries that are used in vehicles. These are lead acid, Nickel Metal Hydride (NiMH) and Lithium Ion (Li-ion) batteries. Nearly all hybrid electric vehicles in production today use NiMH batteries, because they offer the best compromise between energy capacity, size and price, with a specific energy of around 65Wh/kg. These parameters assume a nominal 1 Amphour battery as represented by, Table 1. NiMH batteries also have a fairly rapid recharge time, which is useful for regenerative braking; however, these batteries also have a relatively high self-discharge rate. Lead acid batteries are cheaper but they provide significantly less energy (20Wh/kg) and have a much longer recharge time, which makes them much less attractive for consumer vehicle use. Li-ion has the highest specific power, energy and energy density of the three battery types, and additionally has a very low self-discharge rate. However, these batteries are prohibitively expensive at large sizes, and currently only one production vehicle, the all-electric Tesla Roadster, which has a base price of around $100,000, uses Li-ion batteries. Significantly, gasoline has a specific energy of 12,000Wh/kg; even assuming an internal combustion engine that achieves only 20% efficiency, the total specific energy of such a system is still twice as much as Lithium Ion batteries (90Wh/kg).

**Table 1:** Nominal parameters for 1 Amphour secondary batteries

	Lead acid	Nickel metal hydride	Lithium ion
Specific energy	20-35 Wh/kg	~65 Wh/kg	90 Wh/kg
Energy density	54-95 Wh/L	~150 Wh/L	153 Wh/L
Specific power	~250 W/kg	200 W/kg	300 W/kg
Nominal cell voltage	2 V	1.2 V	3.5 V
Internal resistance	~.022Ω per cell	~.06Ω per cell	~.2Ω per cell
Self-discharge	~2% a day	~5% a day	~10% a month
Recharge time	8h (90% in 1h possible)	1h (60% in 20min possible)	2-3h

Larminie (2003)

### Negative externalities of battery production

There are a number of positive externalities associated with driving hybrid cars instead gasoline cars, including reduced greenhouse gas emissions, reduced air pollution and reduced dependence on foreign oil. These externalities have been discussed elsewhere at great length, so we will not engage in an in-depth discussion of them here. We will instead focus on the less-discussed negative externalities associated with the production of batteries for hybrid cars.

### Issues raised by the production of batteries

The production process is often forgotten in the carbon emission calculation for “clean” energy production or transportation. Indeed, as far as batteries are concerned, the production process itself is energy-intensive and polluting. To give an idea of the scale of energy involved, the production process uses more energy than the batteries are ever going to stock and return during their use. To make 1Wh of capacity in lithium battery, 1.2MJ are needed. To produce the lithium needed, between 0.31MJ and 0.67MJ is used depending on whether the lithium recycled or not. Since a car battery has to have a capacity of about 30KWh, the total energy consumed during production is 56.1GJ.

Moreover, this process is polluting. The U.S. Bureau of Mines estimates that 8 tons of sulphur are produced and emitted for each ton of nickel mined. Lead compounds, such as oxides, are released as particulates during both primary and secondary (recycling) lead smelting operations and during battery manufacture and recycling.

The other issue raised by the production process is the need for natural resources. The outlook for nickel and lithium are outlined below.

**Figure 1:**
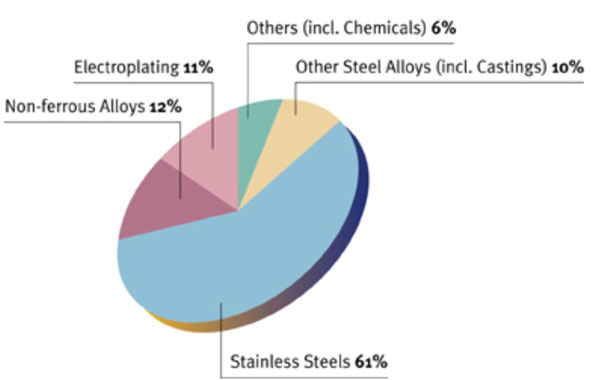
Nickel First Use [5]

The major deposits of nickel are in Canada, Russia, Brazil and China. These are countries that already have major resources, such as oil and gas. Consequently, using nickel instead of gasoline to power vehicles would not substantially change the economic and power equilibriums. A shift to nickel would lessen the dependence on Middle East countries for oil, but it would not create a dramatic shift in which countries currently have natural resources that are considered valuable. Another issue presented by the use of nickel batteries is the competition from stainless steel. Currently around 60% of nickel is used for this purpose (see Figure 1).

**Figure 2:**
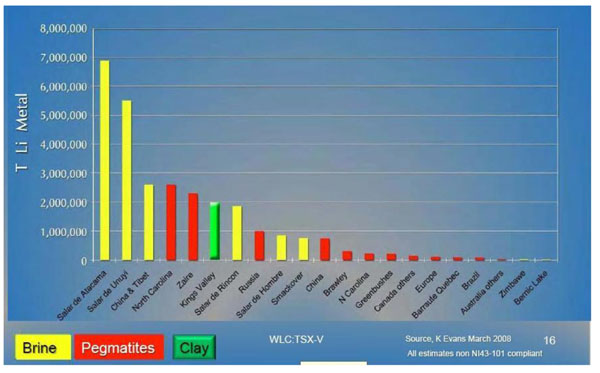
World Lithium Resources [6]

**Figure 3:**
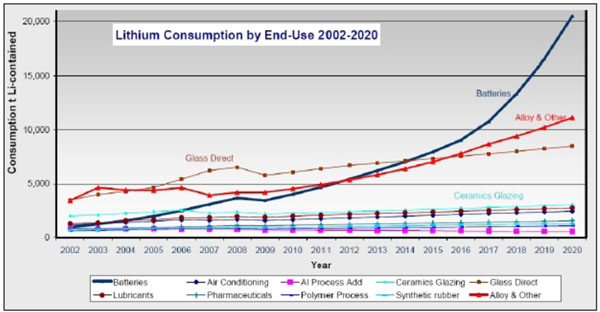
Lithium Consumption by End-Use 2002-2020 [7]

For lithium, the situation is different. Lithium is a very abundant element (the 33rd most abundant) but there are not many places on earth where it is concentrated enough to make mining viable (see Figure 2). There are major deposits in Bolivia, Chile, China and Argentina, countries that are not considered important for oil. As a result, the use lithium instead of gasoline for cars would substantially modify the economic equilibrium. For this reason, some developed countries have tried to maintain the use of nickel technology instead of lithium, even when there is a huge demand for nickel for other purposes besides battery production. Additionally, the overall demand for lithium is rapidly increasing (see Figure 3), and as a result its market price has increased by a factor of ten in five years.

**Figure 4:**
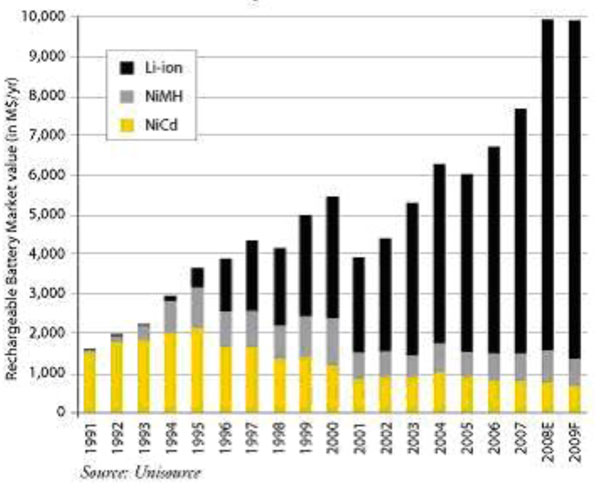
Battery Market Booms

Lithium and nickel demands are increasing, and rocketing demand for batteries (see Figure 4) will only make the situation worse. Battery vehicles do not mean the end of resource dependence, but instead a shift in which resources are important. Other countries’ resources and external price fluctuations will still be necessary to power automobiles.

### Issues raised by the destruction of batteries

Disposal and destruction of batteries raises a number of externality concerns. To make Li-ion batteries, lithium is reacted with nitrogen, oxygen and steam to form a mix of lithium hydroxide, lithium carbonate and lithium nitride. Lithium hydroxide is very corrosive and can cause skin burns and pulmonary edema. For nickel, the problem is similar. Nickel in contact with nitrogen, oxygen or steam produces potassium hydroxide which is very dangerous. Nickel-based batteries can endanger aquatic life since non-recycled batteries are often discarded in the ocean. In the U.S. the recycling rate is around 95% (96% for lead acid batteries).

There is a need for recycling infrastructure and incentives. There are several issues raised by the recycling process. First, the entirely of the battery cannot be recycled. For lithium ion batteries, 90-98% of the material can be recycled. While this constitutes a large portion of the battery, there is still the question of what to do with the remaining 2-10%.

Another issue lies in the assignment of responsibility for recycling. Currently, regulations tend to make the producer responsible for the recycling process. For instance, the Toyota Prius and Honda Civic are equipped with Panasonic batteries. When a battery is not working, it is sent back to Panasonic’s plants in Japan and Panasonic takes care of the recycling. The recycled materials are used to manufacture new batteries. Companies to whom the batteries are sent back either dilute them in their stock, which is not polluting, or burn them. In the absence of government regulations, if the recycling process is not carried out properly, it can be very polluting.

New recycling methods have been implemented, but remain expensive. Thus, for the recycling process to be efficient, it should be centralized. This is currently the case in Illinois where the public service RBRC, with its Call2Recycle program, centralizes the recuperation of batteries and sends them to three recycling centers on the continent. This program is not completely free of problems because the batteries have to be transported, which results in greenhouse gas emissions. Additionally, accidents during the transportation process could result in a spill of toxic components of the batteries. Consequently, there are still a number of issues surrounding the recycling of HEV batteries.

### Energy policy frontiers

There are two major goals that can be achieved by policy changes and implementation regarding battery fuel energy. One is to reduce U.S. dependence on oil, particularly on foreign oil imports from the Persian Gulf. The second is to reduce carbon dioxide emissions by using less oil and more battery fuel energy. To achieve these goals, there are both domestic and foreign policies that could encourage progress.

On the domestic side, there is very little that is currently being implemented to aggressively encourage battery energy advancement. Peter Fontaine writes in *The Electricity Journal* that the government should aggressively encourage battery fuel advancement via provision of tax benefits for using battery fuel, and creation of more funds and monetary awards for companies that advance battery technology. Tax credits are, in fact, currently being given for hybrid car users; the Energy Policy Act of 2005 included a provision for tax credits ranging from $250 to $3,400 per vehicle, in effect from January 2006 through December 2009. However, there is a limit of approximately 60,000 vehicles per manufacturer that can qualify for this tax credit. Salvatore Lazzari, a specialist in public finance resources, science and industry division writing for the U.S. Congress, explains that this limit was instituted in order to limit the benefits accrued due to imported hybrid vehicles, which currently outnumber domestically manufactured hybrid vehicles in the market [3].

Although this limit serves a protective purpose for the domestic market, Peter Fontaine insists that the government should do more. Fontaine argues that it is necessary for the government to more aggressively promote battery fuel so that it can effectively penetrate the current oil-dominated energy market, and encourage CO_2_ emission reduction practices such as cap and trade CO_2_ emissions trading [4]. Fontaine’s concern is validated when we look at current energy policies; despite the inclusion of battery fuel advancement in the American Clean Energy and Security Act, battery fuel is not included as a standard in the Clean Air Act Renewable Fuels Standard [5]. However, cap and trade programs have been practiced on a smaller scale, providing hope that a program could be implemented in a wider region in the future.

There are several implications to the proposed approaches to promoting battery development. Mainly, as Rene Kemp, Johan Schot and Remco Hoogma explain in *Regime Shifts to Sustainability Through Processes of Niche Formation*, the dominant energy regime is difficult to diminish because the oil industry is locked into its niche market. The oil establishment has been solidified over decades of industry prominence and it is resistant to regime change despite aggressive government policy implementations [6]. In addition, the oil industry lobbies for its interests to stay relevant and predominant in the market. Therefore, the government would face strong opposition from oil firms, rendering it difficult to push through significant changes in battery fuel development and implementation.

On the foreign policy side, there are several essential goals that can be achieved through pro-battery policy: reduction of our national dependence on foreign oil and a simultaneous reduction in the oil market’s economic activity on the international level, as well as a reduction in the military costs of protecting key oil-related regions such as the Persian Gulf and the Strait of Malacca. These goals are also importantly related to foreign policy goals such as reducing tension between China and the U.S. over the Saudi Arabian oil market, and reducing arms trade towards Saudi Arabia for the amelioration of oil trade between it and key states such as the U.S. and China.

Achieving these goals allows for a multitude of benefits that would relieve many costly and longstanding international crises. For one, protecting the Persian Gulf and securing sea trade routes for oil such as the Strait of Malacca costs between $70 and $100 billion per year [7]. Reducing dependence on oil would not only relieve the U.S. of this costly burden, but it would also reduce the need for continued U.S. involvement, both diplomatic and militaristic, in the region. At this point, both the U.S. and China import oil from Saudi Arabia, and this causes tension between the three states. If both importing states grew less dependent on oil, this tension would dissipate and the U.S. would have less need to supply Saudi Arabia with arms deals that are primarily serve to appease the oil market with preferential treatment.

Although the U.S. has much to gain from achieving these goals, there are a number of difficult political hurdles. For one, it would be partially beneficial to encourage a race towards battery technology enhancement between the U.S. and China. This would also encourage China to be more energy-independent and consequently less dependent on Saudi Arabia. However, international energy crisis researchers such as Michael Klare suggest that alternative fuels will not actively advance unless they soon become more profitable to pursue than the preexisting energy mainstays [8]. In addition, it is unclear how soon the U.S. would be able to withdraw from its Persian Gulf involvement even if alternative energy forms were to relieve the need for oil; just as the oil industry is solidly established and resistant to regime change, the U.S. involvement in the Persian Gulf has become entrenched. With the many political and military involvements that have conspired between the U.S. and the Middle East since the 1980s, the U.S. cannot easily withdraw its involvement quickly while the Middle Eastern states remain unstable. Though the U.S. has much to gain from improvements in battery technology, there are still some obstacles that stand in the way of the achievement of these goals.

### Future prospects for battery technology

Lithium-ion batteries are the future of car batteries. As discussed earlier, Li-ion batteries have better specific power, energy and energy density than lead acid and NiMH batteries, along with a very low self-discharge rate. These characteristics make them the best choice for use in vehicles; however, there are still a number of limitations that have prevented car manufacturers from adopting Li-ion batteries. These limitations explain why there is currently only one commercially available vehicle that uses Li-ion batteries, the all-electric Tesla Roadster sports car, which, at a base price of over $100,000, is more of a specialty car than a replacement for the average internal combustion passenger vehicle.

Though Li-ion batteries have been widely used in laptops, cell phones and other consumer electronics, batteries of the size necessary to power an automobile face a number of limitations. These limitations can be summed up in four categories: safety, cost, life, and performance over a wide temperature range. Safety concerns are primarily centered on thermal runaway, a positive feedback mechanism that results in overheating, which can ultimately cause sealed-cell batteries to explode. The costs of Li-ion batteries for vehicles are currently prohibitively high, as is apparent from the price of the Tesla Roadster. Additionally, Li-ion batteries need to have a longer life if they are to be used in vehicles. Every time that a Li-ion battery is recharged, deposits form in the electrolyte that inhibit lithium ion transport, which decreases the capacity of the cell. This means that as Li-ion batteries age, they are able to hold less charge. Finally, the poor performance of Li-ion batteries at temperatures below freezing limits the widespread deployment of these batteries.

The majority of current research efforts into the future of Li-ion batteries have been undertaken by government agencies or with government support. The principal government research program is the Vehicle Technologies Program (VTP), housed within the Department of Energy’s Office of Energy Efficiency and Renewable Energy. VTP is a collaborative research initiative that aims to develop advanced transportation technologies that would improve vehicle fuel efficiency and reduce petroleum dependence, helping the U.S. to achieve transportation energy security. The Program’s budget for financial year 2009 was $273 million, and an additional $60 million has been requested for FY 2010 [9]. VTP has five major strategic areas: vehicle electrification, high-efficiency engines, advanced lightweight materials, fuels and lubricants, and deployment and education. Vehicle electrification efforts involve research into lowering battery cost while increasing battery performance and life. VTP collaborators include industry leaders, national laboratories, universities, and state and local governments.

One of the industry partners in the VTP program is the U.S. Council for Automotive Research (USCAR), which includes Chrysler Corporation, Ford Motor Company and General Motors Corporation. USCAR and DOE have established two specific goals for battery technologies: to reduce battery cost to $20/kW and extend calendar life to 15 years [10]. Argonne National Laboratory is another VTP partner working to meet these goals. Argonne Lab, located outside of Chicago, hosts the Transportation Technology R&D Center, which is leading DOE’s R&D program on Li-ion batteries for transportation applications, and addressing the limitations of Li-ion batteries. Argonne is working to improve the safety of Li-ion batteries by examining the thermal properties of the battery materials and other components, and experimenting with new electrode materials that produce less heat, as well as electrolyte additives that retard flammability within the cells. In order to reduce battery costs, Argonne researchers have developed software tools that can be used to design Li-ion batteries for transportation applications. Taking into account the materials used to make the battery and the production rate, these tools are then used to estimate battery costs, without needing to actually construct the battery prototype. Argonne Lab is also working to improve the calendar life of batteries by using advanced diagnostic techniques to conduct accelerated cell aging studies. These studies allow scientists to better understand the mechanisms that affect power and capacity loss over time, and to develop more stable materials for batteries that improve life. These same diagnostic modeling studies also allow researchers to determine causes of poor battery performance at low temperatures [11].

Aside from the Vehicle Technologies Program, the federal government has also supported the development of vehicle batteries through legislation, most recently through the American Recovery and Reinvestment Act of 2009. The Recovery Act designated $2.4 billion for domestic manufacturing of automobile batteries and related components. From these funds, $250 million was granted to A123 systems, one of the few American Li-ion battery makers, to build a Li-ion battery factory in Michigan [12].

## Methods

### Cost-benefit analysis of cars – gasoline versus hybrid

Aside from putting forth an argument for environmental friendliness, car salesmen and corporate executives in the business of selling HEVs have claimed that after taking into account all costs throughout the life of the vehicle, it is more economical to own hybrid cars than gasoline cars. Some of these individuals have gone so far as to attempt to show this quantitatively by adding up the future costs of both types of cars.

Although many people may believe in the numbers they are shown by car salesmen, the numbers themselves are essentially meaningless calculations unless the future costs are discounted to their present values. To illustrate this more clearly, we will take a hypothetical example with two periods. Car A costs 100 and car B costs 140 in period one. In period two, the cost of maintaining car A is 50 whereas car B has no maintenance costs. By simple math, similar to what car salesmen use to “prove” that hybrid cars are cheaper than gasoline cars, car A costs a total of 150 and car B costs a total of 140. Hence, car A is more expensive than car B.

However true this may be true in a hypothetical world without financial markets and opportunity costs, it is definitely false in the real world. Continuing from the above example, we will add in the option for the buyer to either buy one of the two cars, or to put his money in the bank with a 30% interest rate. This means that although a buyer purchasing car A is obligated to pay 50 in the future, the interest returns from the bank means in order to pay the maintenance costs in the future, the buyer only has to have about 38 in the present (period one), because that 38 will be worth 50 in the future (period two).

Taking into account the 30% rate of return by the bank in this example, the present costs when the car is purchased in period one is 138 for car A and 140 for car B, which shows that car A is cheaper when costs are calculated with correct quantitative analysis. Using a simple example with two extra variables – time and a rate of return – already yields completely different results. By the same token, the simple math used by car salesman to show that hybrid cars are cheaper than gasoline cars deviates far from the reality of many more time periods and cost variables, which leads us to the two questions this paper attempts to answer:

(1) Are the simple math calculations used by car salesmen a marketing gimmick?

(2) Do the economic benefits outweigh the costs for hybrid cars?

### Setup

**Figure 5:**
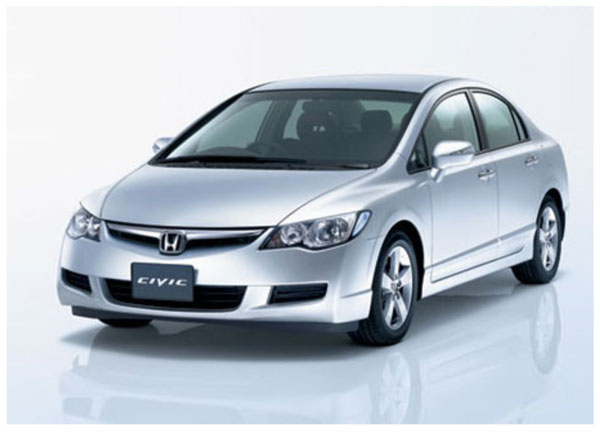
The Honda Civic

To achieve an analysis with significant results, we first narrowed the scope of our study in order to prevent making farfetched assumptions. Our study will focus on the United States as the geographic region, because of the abundance of economic data available. The timeframe of our study will be 5 years (14 years taking into account future costs). As with all models, a longer time frame allows more future uncertainties. Furthermore, 14 years of data can successfully answer the two main questions of our study. Our study will focus on the Honda Civic (Figure 5). The Honda Civic is currently and has historically been one of the most popular cars in the world, but more importantly it has both a gasoline and hybrid model. Although not all specifications for the two versions are exactly the same, this is the closest comparison available to allow for a fair test.

To quantitatively set up the cost-benefit analysis, we start from the “with versus without” principle – the “with” being hybrid and the “without” being gasoline cars. Since we know the costs for the consumer, the goal is to minimize costs.

The logic can be summarized as follows:

• With (hybrid) versus without (gasoline)

=> New state (hybrid) versus original state (gasoline)

• Goal – minimize cost

=> Cost of new state < cost of original state

=> (Cost of original state) - (cost of new state) > 0

=> (Cost of gasoline) - (cost of hybrid) > 0

• Calculate – net = (C_G_) – (C_H_)

As long as the net is positive, this means hybrid cars are lower in cost and carry positive economic benefits.

Note that our cost-benefit analysis will not quantitatively consider the distribution of benefits but focus on total size of the benefits to society. This point will be revisited and further elaborated in the next section.

### Assumptions

The five main assumptions of the cost-benefit analysis model in this case study are as follows:

i) Consumer habits –

• Previous studies have estimated that American consumers purchase new cars every 8 to 10 years on average. We will assume this to be 10 years, which is on the higher end, for two main reasons. First, the recent economic crisis has increased savings and decreased the marginal propensity to consume for the average American. Secondly, technology tends to extend the lifespan of most goods.

• Previous studies also show that the average distance driven per year on every American car is 12,000 to 15,000 miles. For our model, we will assume the median of this range, 13,500 miles per year.

ii) Producer habits –

• As previously discussed, we are focusing on the increase in net benefits to the whole of society (i.e. the total size of the pie) rather than distribution of these benefits. Hence, we will assume that the profit margins expressed in percentages for both the gasoline and hybrid car are equal. If profit margins are not assumed to be equal, firms will essentially subsidize the initial purchase price of the car selling at a lower profit margin. Since (total utility to society) = (producer surplus + consumer surplus), an erosion of firms’ profits equates to a payment transfer from producer to consumer rather than an increase in overall value to society.

• Although this assumption may seem unrealistic, which is true if one analyzes margins on a single year basis, taking the average of the purchase prices and costs of production over the years for each vehicle, and then calculating the profit margins should yield almost identical results. Historically the profit margins for hybrid vehicles have indeed been lower than gasoline cars, but looking forward, this trend is likely to be reversed, as Japanese car manufacturers project that by 2020, they will be able to cut costs of hybrid cars by up to 67 percent. At the same time, Toyota has claimed that absolute profit margins for their hybrid cars should be equal to gasoline cars from 2010 onwards. Toyota has further claimed that they will be selling 100% hybrid cars by 2020. Since hybrid cars entered the market in a profound way around year 2000, our model, which calculates its values from 2010 onwards, should be in the middle transition period where hybrid vehicles’ profit margins are approaching and surpassing those of gasoline vehicles. This implies that our profit margin assumption is reasonable.

iii) Discount rate –

• Our model will use a discount rate of 7%, the suggested private sector rate of return for models projecting less than 25 years by Professor George Tolley (Economics Department, University of Chicago). As a majority of the firms pioneering hybrid vehicles are extremely profitable and innovative companies, using the private sector rate of return is a more accurate assumption than a risk-free rate of return.

iv) Gasoline prices –

**Figure 6:**
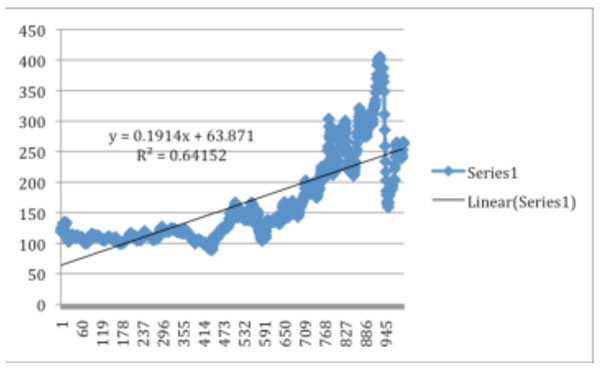
Week (1990 onwards) vs. Cents per gallon

• We have applied linear regression analysis to gasoline prices from 1990 onwards to yield the following trend and regression equation in Figure 6.

• Although both the above regressions have statistical significance based on their R^2^ coefficients, the uncertainty and volatility of oil prices play a major role in calculating the cost of cars, so we have incorporated two additional gasoline price scenarios. By observing how these alternative scenarios affect the results, we are able to account for our model’s sensitivity to gasoline prices.

v) Linear nature of technology –

• Our model assumes the improvement of technology over time to be linear. This cost-benefit analysis model does not need or want to make any judgments on whether technology displays increasing or decreasing returns to scale, nor to spur a philosophical debate (e.g. feasibility of technological singularity).

• Given the short timeframe of 14 years, applying a linear model is not unreasonable even if technology is forever exponential in return.

• The first technological assumption is the decrease in manufacturing and maintenance costs. We can analyze this via the initial price of the car, since firms use the projections of both costs to determine price. It is worthwhile to note that currently, maintenance costs, particularly for hybrid cars, are included in the car’s warranty as part of the purchase. The initial purchase price is the first component of the costs associated with owning a car. Based on the data for gasoline vehicles from year 2000 onwards and Toyota’s projection of decreasing production costs by over 60% 10 years from now, our model assumes prices of gasoline cars to fall linearly at 1% annually net of inflation leading to a total of 5% fall, and the prices of hybrid cars to fall linearly at 2.5% annually net of inflation leading to a total of 12.5% fall. We have selected these conservative assumptions, as there is not adequate and sufficiently significant data to estimate how quickly hybrid cars’ costs will fall. We do not believe this assumption will undermine the results, because future stream of costs is the main issue at stake. This conservative assumption will further add to our conclusions.

• The second technological assumption and the second component that factors into the lifetime cost of the vehicle is the fuel economy – EPA’s miles per gallon (MPG) of the vehicle. Besides improved gasoline combustion by the engine and generation of electricity for the hybrid car, the easiest method to increase MPG is to make the entire vehicle lighter. This is usually done by taking away enhanced features of the modern car such as comfort and performance. What complicates projections for MPG is its relationship with oil prices. Taking our Honda Civic as an example, the fuel economy improved from the upgrade of the 03/05 to the 06/08 models for both the gasoline and hybrid cars. However, the bust of the oil bubble has actually led to a fall in the average fuel economy of passenger vehicles. This is obviously not due to technology moving backwards, but because of lower oil prices, newer cars have been designed with little attention to fuel economy, and often include enhanced features resulting in heavier vehicles.

• Table 2 below shows the improvement in MPG from every cycle of new model releases. Disregarding the latest release because of the issue with the bust of the oil bubble mentioned previously, we will assume that three cycles of upgrades will be available in the next 14 years. Averaging over 14 years yields 0.63% and 1.13% linear annual increases in MPG for gasoline and hybrid cars respectively. Historical business cycles show that it is safe to assume an economic boom to take place after a bust like the one that we have just experienced, so we can also expect oil prices to increase steadily within this period of time, substantiating the assumption for an increase in MPG.

**Table 2:** Fuel economy of the Honda Civic

Honda Civic EPA’s MPG	Gasoline	Hybrid
2003-2005	34	47.5
2006-2008	35	50
1 cycle	2.94%	5.26%
2 cycle	5.88%	10.52%
3 cycle	8.82%	15.78%
Averaged over 14 years	0.63%	1.13%

## Results

The following outlines the main building blocks of our model and the steps we took to derive the results:

(1) Gasoline price –

Using the regression trend, *y* = *0.1004x - 199.45*, we obtained the projected gasoline prices from year 2010 to 2023. Adding in the sensitivity of this variable, we projected two additional cases – gasoline prices 25% higher and 25% lower than the regression trend’s projection. These projections yield the results in Table 3.

**Table 3:** Gasoline price projections

Year	2010	2011	2012	2013	2014	2015	2016
Gasoline prices 1	2.354	2.4544	2.5548	2.6552	2.7556	2.856	2.9564
Gasoline prices 1.25	2.9425	3.068	3.1935	3.319	3.4445	3.57	3.6955
Gasoline prices 0.75	1.7655	1.8408	1.9161	1.9914	2.0667	2.142	2.2173

Year	2017	2018	2019	2020	2021	2022	2023

Gasoline prices 1	3.0568	3.1572	3.2576	3.358	3.4584	3.5588	3.6592
Gasoline prices 1.25	3.821	3.9465	4.072	4.1975	4.323	4.4485	4.574
Gasoline prices 0.75	2.2926	2.3679	2.4432	2.5185	2.5938	2.6691	2.7444

(2) Purchase price of car –

Applying the assumptions (v) of the annual price decreases of the cars, 1% and 2.5% for Honda Civics’ gasoline and hybrid versions respectively yields the following projections in Table 4 for the price of the cars in the next 5 years.

**Table 4:** Purchase price projections

Year	2010	2011	2012	2013	2014
Gasoline $	16455	16290	16125	15961	15796
Hybrid $	23800	23205	22610	22015	19813

(3) Fuel economy of car –

Similarly, applying the MPG assumptions which projects annual increases in MPG of 0.63% and 1.13% for Honda Civics’ gasoline and hybrid version in the next 14 years, we obtain the following projections, reflected on Table 5 below, on absolute increases in MPG.

**Table 5:** Fuel economy projections (MPG)

Year	2010	2011	2012	2013	2014	2015	2016
Gasoline MPG	29.000	29.182	29.365	29.548	29.730	29.913	30.096
Hybrid MPG	42.000	42.462	42.924	43.386	43.848	44.31	44.772

Year	2017	2018	2019	2020	2021	2022	2023

Gasoline MPG	30.278	30.461	30.644	30.820	31.009	31.192	31.375
Hybrid MPG	45.234	45.696	46.158	46.620	47.082	47.544	48.006

(4) Cost of gas –

With the projected MPG, we first calculated the projected annual cost of gas by applying the data from the assumptions and projections into the following equation.

Applying this equation to every year from 2010 to 2023, we obtain the cost of gas per year with the two additional scenarios – 25% above and below, reflected on Table 6, projected gas prices – for sensitivity analysis.

**Table 6:** Gasoline cost scenarios

Year	2010	2011	2012	2013	2014	2015	2016
Gasoline gas cost	1095	1135	1174	1213	1251	1288	1326
Hybrid gas cost	756	780	803	826	848	870	891
Gasoline +25%	1369	1419	1468	1516	1564	1611	1657
Hybrid +25%	945	975	1004	1032	1060	1087	1114
Gasoline -25%	821	851	880	909	938	966	994
Hybrid -25%	567	585	603	620	636	653	669

Year	2017	2018	2019	2020	2021	2022	2023

Gasoline gas cost	1363	1399	1435	1471	1506	1540	1574
Hybrid gas cost	912	933	953	972	992	1011	1029
Gasoline +25%	1704	1749	1794	1838	1882	1925	1968
Hybrid +25%	1140	1166	1191	1215	1240	1263	1286
Gasoline -25%	1022	1049	1076	1103	1129	1155	1181
Hybrid -25%	684	700	715	729	744	758	772

Using these cost projections, we discount each year’s cost of gas to obtain the present value of costs by the following equation.

Using the above equation, we obtain the total costs of gas for individuals purchasing cars in the next 5 years, reflected on Table 7 below.

**Table 7:** Discounted gasoline price scenarios

Year	2010	2011	2012	2013	2014
Gasoline gas total cost	9374	9658	9938	10214	10488
Hybrid gas total cost	6353	6517	6678	6835	6989
Gasoline total +25%	11717	12072	12422	12768	13110
Hybrid total +25%	7941	8146	8347	8544	8736
Gasoline total -25%	7030	7243	7453	7661	7866
Hybrid total -25%	4765	4888	5008	5126	5242

(5) Real cost of car = purchase price + total cost of gasoline is reflected below in Table 8:

**Table 8:** Real cost of cars

Year	2010	2011	2012	2013	2014
Gasoline gas real cost	25829	25948	26063	26176	26284
Hybrid gas real cost	30153	29722	29288	28850	26803
Gasoline real +25%	28172	28362	28548	28729	28906
Hybrid real +25%	31741	31351	30957	30559	28550
Gasoline real -25%	23485	23534	23579	23622	23663
Hybrid real -25%	28565	28093	27618	27141	25055

(6) Final results –

Recall the setup of this cost-benefit analysis,

• With (hybrid) versus without (gasoline)

=> New state (hybrid) versus original state (gasoline)

• Goal – minimize cost

=> Cost of new state < cost of original state

=> (Cost of original state) - (Cost of new state) > 0

=> (Cost of gasoline) - (Cost of hybrid) > 0

• Calculate – Net = (C_G_) – (C_H_)

We find the net by subtracting the real cost of gasoline cars by the real cost of hybrid cars, which is reflected in Table 9 below.

**Table 9:** Net costs of purchasing a hybrid car instead of a gasoline car

	2010	2011	2012	2013	2014
Net 1	-4324	-3774	-3224	-2674	-518
Net +25%	-3569	-2989	-2409	-1830	356
Net -25%	-5079	-4559	-4039	-3519	-1393

## Conclusions

The result of this analysis show that gasoline vehicles currently remain cheaper than hybrid vehicles. In other words, the salesman who touts the economic advantages of hybrid cars is incorrect. This is true in all three of our gasoline price scenarios (baseline, high and low), though the results are sensitive to gas prices, so the price differential is smallest in the high-price scenario. However, even if oil prices do not increase dramatically, there is strong economic argument to support the investment and consumption of hybrid cars.

Under our baseline gas price scenario, for a car purchased in 2010, the total lifetime costs of the hybrid are about $4,324 higher than those of the gasoline car. For a car purchased in 2014 the differential is $4,200, in 2012 $4,080, in 2013 $3,960, and in 2014 $3,840.

However, it is evident that going forward, the decrease in cost of production for hybrid vehicles and increasing oil prices will close the gap. Our results show that it is likely that after 5 years, using hybrid vehicles will be cheaper, and will in effect yield a positive net benefit to society.

It is important to note that our analysis only takes into account the price of the car to the consumer. These costs do not represent the total cost of the car to society. There are a number of externalities that could significantly impact the total social cost of the car. These externalities can be divided into four categories: environmental, industrial, R&D and other social externalities. We have already addressed some of the negative environmental externalities of the production of batteries for HEVs. The negative environmental externalities of gasoline cars include particulate matter, greenhouse gas emissions, and foreign oil dependence. Particulate matter air pollution has been linked to a number of negative health impacts, including asthma, chronic respiratory illness, heart attacks and premature mortality. Treating these illnesses imposes large health costs on society, and these costs are even higher if the value of life is taken into account. Additionally, passenger vehicles are responsible for 26.5% of U.S. greenhouse gas emissions; though the cost of carbon emissions is difficult to quantify, the cap and trade system that has been proposed in the recent climate bills in Congress would place a definite per-unit price on CO_2_ emission.

We have also briefly addressed the externalities of an increase in hybrid production on other industries. An increase in lithium demand for the production of Li-ion batteries could result in a power shift in international trade, since the countries with large deposits of lithium are not the same countries which currently possess important natural resources. Additionally, increased reliance on HEVs would decrease the importance of the oil industry in the global market. Reduced U.S. dependence on foreign oil from Canada and especially the Persian Gulf would allow the U.S. to withdraw much of its military protection that it has been maintaining despite high costs. Withdrawing from the Persian Gulf region could diminish the rising political tension that the U.S., China and Saudi Arabia have been fostering over the region's oil market. Decreased U.S. dependence on Saudi Arabian oil could consequently improve the U.S.’s relations with China.

There are other externalities associated with hybrid car production and development that are harder to quantify. For one, there are the research and development costs that go into producing advanced batteries. However, these costs need not be included in an accounting of the cost of a hybrid vehicle, as R&D costs are sunk costs. It would be unreasonable to attempt to include all the costs of technology development reaching back to Henry Ford, and similarly it is not necessary to account for more recent R&D costs. Another externality is the psychological “feel-good” benefit that consumers may derive from driving a hybrid car that is “greener” than a traditional gasoline car. Driving a hybrid car instead of a gasoline car also imposes a positive externality of time saved, since less time would be spent at the gas pump, refueling the car.

Given the positive externalities of driving hybrid cars and the negative externalities of driving gasoline cars, it seems reasonable that the government would work to support the adoption of hybrid cars. The Department of Energy’s Vehicle Technologies Program does this, to an extent; given volatile gas prices and the possibility of a carbon price, it is possible that in the future we will see more aggressive promotion.

Hybrid-electric passenger vehicles are poised to impact U.S. markets in a significant way. HEVs have the potential to increase America’s energy independence by reducing U.S. foreign oil dependence, as well as to significantly decrease greenhouse gas emissions and consequently mitigate global climate change. Battery technology is one of the largest obstacles in the deployment of HEVs. The resources required to produce batteries could shift the global power structure, and the recycling of these batteries is another issue that is largely unregulated. Technological limitations tend to render batteries prohibitively expensive, increasing the price of hybrid vehicles over comparable gasoline-powered vehicles. However, joint government-industry research efforts into methods of reducing these battery costs and improving battery technology show great promise. Though costs to the consumer will be higher over the next five years for hybrid cars than for gasoline cars, hybrid cars will soon break even with gasoline cars, spurring greater market penetration by HEVs and further incentivizing advanced battery research.

## Competing interests

The authors Heather Lewis. Haram Park and Marion Paolini have no competing interests.

## Authors' contributions

HL carried out the background study on future battery industry prospects and its technological frontiers, and approved the final manuscript. HP carried out the historical and foreign policy research and analysis, and drafted the final manuscript for approval. MP carried out the study on the issues raised by the use and production of batteries.
